# Juxta-Articular Synovial Hemangioma of the Ankle: A Rare Benign Tumor

**DOI:** 10.7759/cureus.81596

**Published:** 2025-04-02

**Authors:** Anmol Kankane, Ashish Rustagi, Utkarsh Jain, Loveneesh Krishna, Jatin Talwar

**Affiliations:** 1 Orthopaedics, Vardhman Mahavir Medical College and Safdarjung Hospital, New Delhi, IND

**Keywords:** ankle and knee arthroscopy, ankle swelling, ankle tumors, benign tumor, hemangioma treatment, joint swelling, rare soft tissue tumors, synovial hemangioma

## Abstract

Synovial hemangioma is a rare benign tumor with abnormal proliferation of blood vessels and is more common at the knee than at the ankle. We present the case of a 14-year-old male with an eight-year history of a progressively enlarging, painful swelling in the right ankle. Radiological investigations, including MRI, suggested a juxta-articular synovial hemangioma, which was confirmed on histopathology following ultrasound-guided biopsy. Initial management involved angiographic embolization; however, due to persistent symptoms, surgical intervention was performed. The patient underwent an en-bloc extracapsular excision, leading to full recovery with restored joint function and pain-free weight-bearing within six weeks postoperatively.

## Introduction

Synovial hemangioma is a rare benign soft tissue tumor of the joint cavity. They comprise around 0.07% of all soft tissue tumors and fewer than 1% of all excised hemangiomas [1], with around 60% arising in the knee joint [2]. It consists of abnormal proliferation of blood vessels arising from the synovium of joints, bursae, and tendon sheaths. It is typically an intra-articular mass lined by synovium and may be pedunculated or diffused [3,4]. Rarely, it can also be juxta-articular (outside the joint capsule but in close relation to it) or intermediate [1]. Clinically, it is a very slow-growing, painful swelling (seldom painless) without any eventful history, rendering a delayed presentation from the onset of symptoms. They may include recurrent hemarthrosis, chronic joint pain, swelling, and mechanical symptoms, such as locking or clicking, which over time can cause secondary degeneration of the joint. The diagnosis is preliminarily radiological with final confirmation on histopathological examination.

## Case presentation

Case history

A 14-year-old boy presented to the orthopedic outpatient clinic with a chief complaint of a swelling in his right ankle for the past eight years, which was initially neglected, insidious in onset, and minimally but gradually progressive in size for the last year. The swelling was now accompanied by intermittent dull aching pain, mostly while walking, but did not significantly hamper his day-to-day functions. There was no history of trauma or any similar joint swelling at present or in the past.

**Figure 1:**
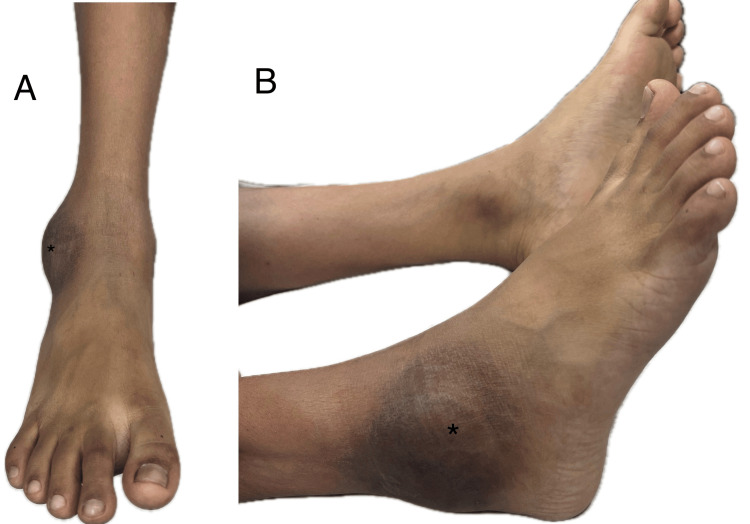
Clinical picture of the swelling on the right ankle viewed from the front (A) and side (B). Swelling labelled with an asterisk (*).

On examination, there was a 7 × 5 × 2 cm well-circumscribed swelling on the lateral aspect of his right ankle just anteroinferior to the lateral malleolus (Figures 1A, 1B). There were no skin changes apart from a slight hyperpigmentation. The swelling was firm, non-fluctuant, and free from the overlying skin but adhered to the underlying structures, making it non-mobile with only mild tenderness. There was a slight painful limitation of dorsiflexion and eversion terminally.

**Figure 2:**
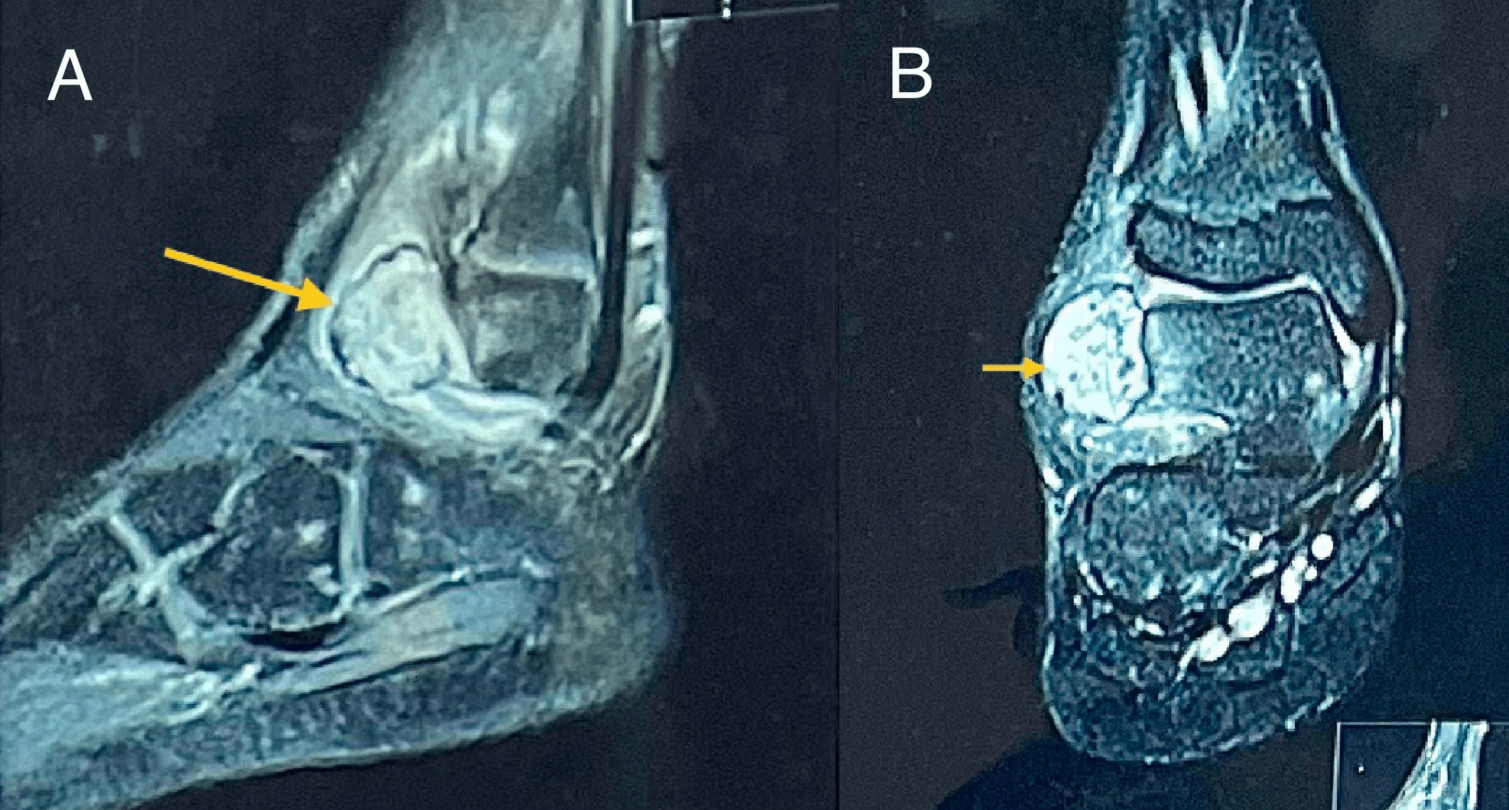
MRI with saggital (A) and coronal (B) sections showing a well-defined soft-tissue intensity lesion in the subcutaneous plane over the ankle joint anterior to the lateral malleolus. The lesion appears to be abutting the lateral aspect of the talus and causing scalloping of its lateral cortex. Adjacent bone marrow edema can be seen on PDFS sequences. PDFS: proton density with fat suppression

MRI of the ankle gave a provisional diagnosis of a synovial hemangioma (Figures 2A, 2B). A fine-needle aspiration was performed which yielded frank blood. An ultrasound-guided biopsy was planned, which was consistent with the MRI of a juxta-articular synovial hemangioma of the ankle.

**Figure 3:**
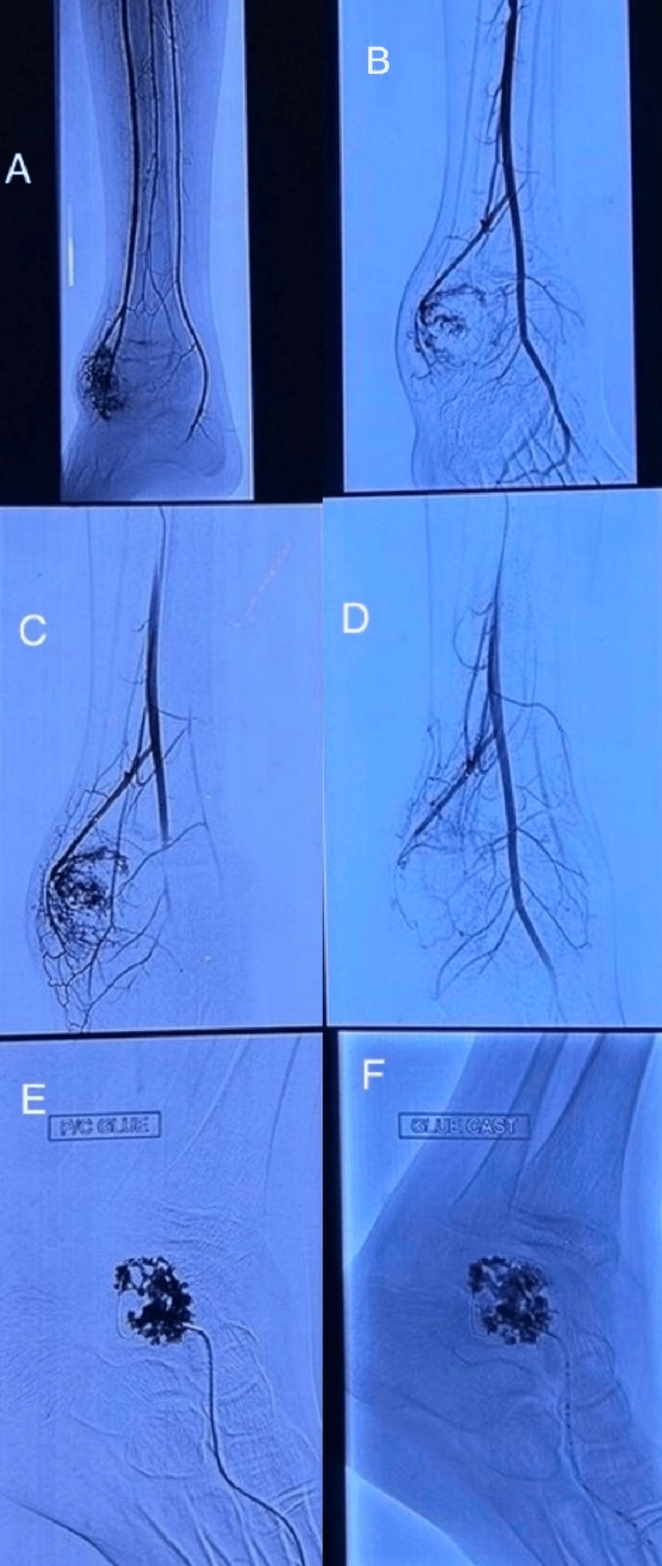
Fluoroscopic images showing steps (A-F) of angiography and embolization of the feeder vessel of the tumor using PVA particles and a combination of cyanoacrylate glue and lipiodol (33%). PVA: polyvinyl alcohol

**Figure 4:**
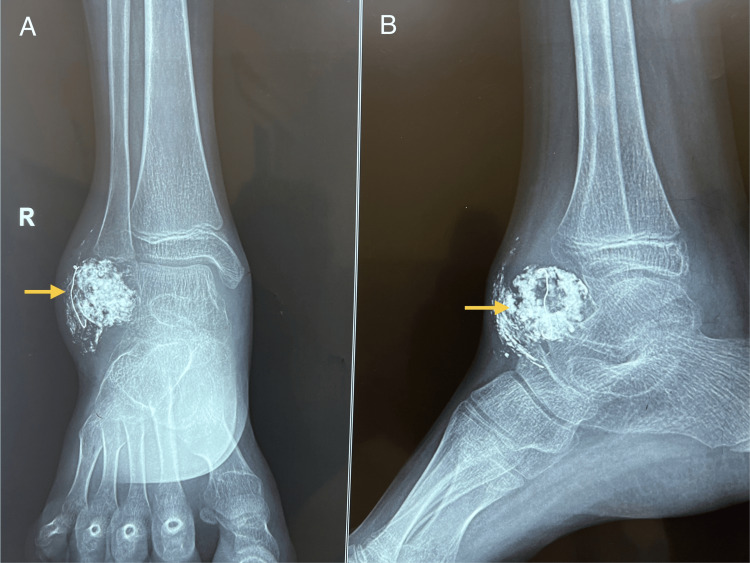
Post-angio-embolization anteroposterior (A) and lateral (B) radiographs of the ankle showing calcification (phlebolith) in the soft tissue tumor with the embolized feeder vessel.

**Figure 5:**
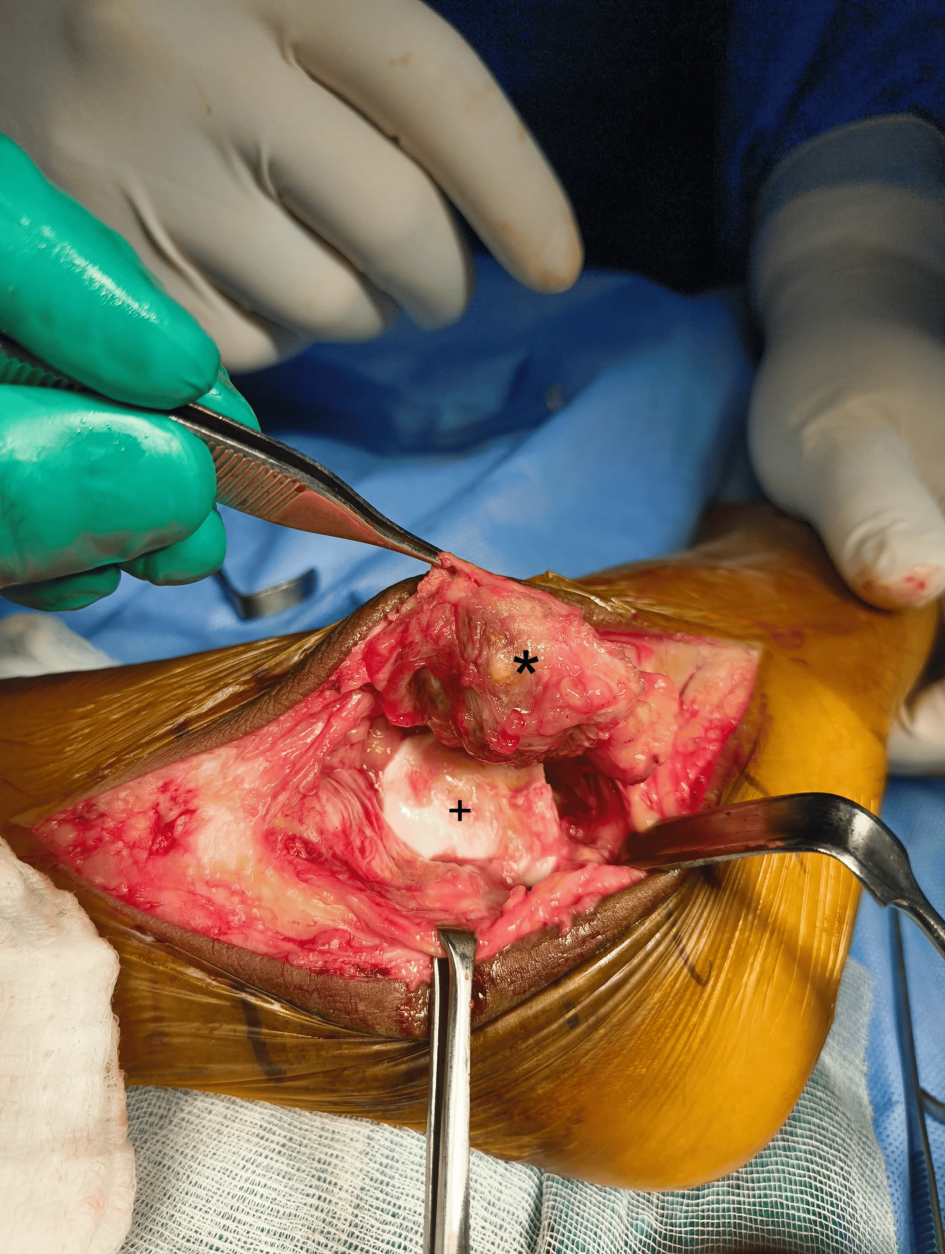
Intraoperative image showing the tumor and scalloping of the talus underneath. Tumor labelled with an asterisk (*) and talus labelled with a plus (+).

The patient underwent angiography and embolization of feeding vessels by the Interventional Radiology team using polyvinyl alcohol particles and a combination of cyanoacrylate glue and lipiodol (33%) under fluoroscopic guidance. The steps of the procedure can be seen in Figures 3A-3F. Post-angio-embolization radiograph of the ankle showed calcification (phlebolith) in the soft tissue tumor with embolized feeder vessel (Figures 4A, 4B). The patient followed up in the outpatient department for three months, but there was minimal to no decrease in the size of the swelling, following which a plan for tumor excision was made after due deliberation with the pathologist and radiologist. An en-bloc extracapsular excision of the hemangioma was performed under tourniquet control via a lateral approach to the ankle under general anesthesia and caudal block. Intraoperatively, a pigmented soft tissue mass was seen arising from the anterolateral ankle joint capsule abutting and scalloping the talus (Figure 5). Postoperatively, the biopsy features were consistent with synovial hemangioma right ankle.

The postoperative period was uneventful, and the patient was allowed weight-bearing as tolerated. Sutures were removed at two weeks, and he had a full range of motion at the ankle with painless weight-bearing on the right lower limb at the end of six weeks.

Pathological findings

**Figure 6:**
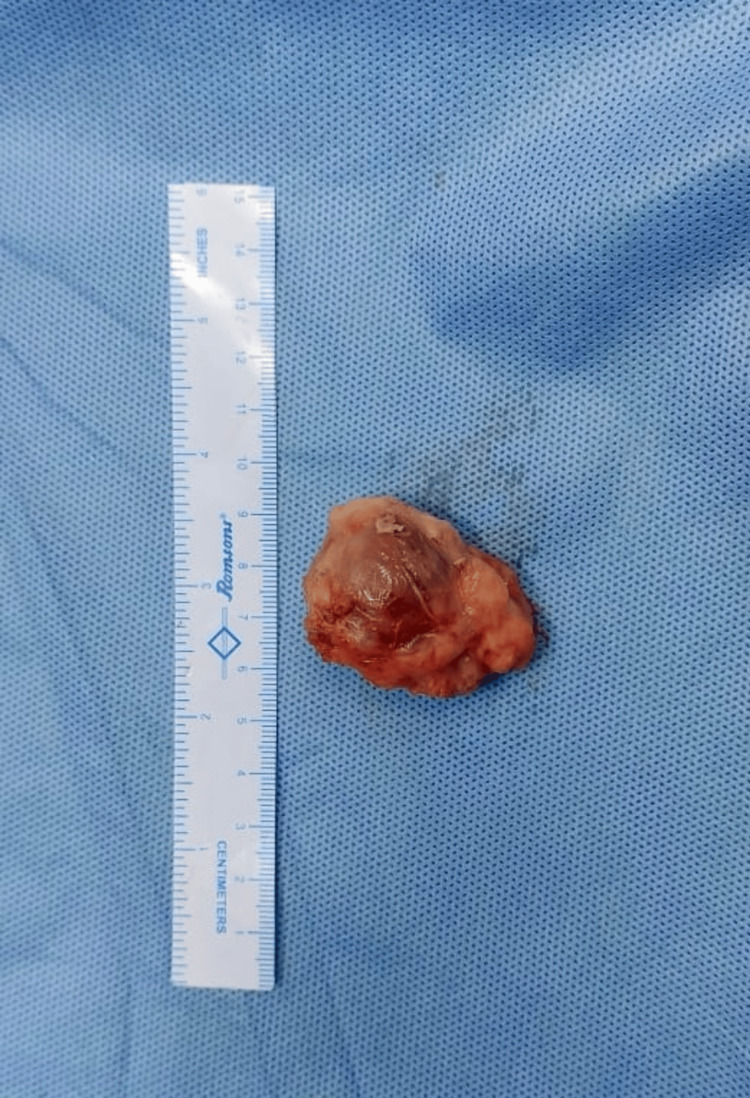
Gross morphology of the excised tumor with a fibrofatty outer surface measuring 4 × 3 × 1.5 cm.

**Figure 7:**
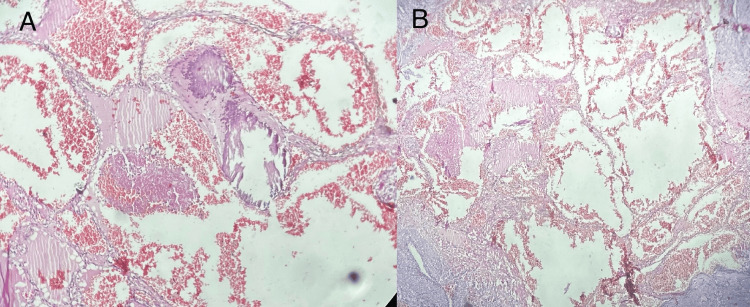
Microscopic examination of the tumor showing sections (A and B) with fibrocollagenous tissue showing dilated, anastomosing, thin-walled capillaries lined by flattened endothelium.

Grossly, a single fibrofatty tissue piece measuring 4 × 3 × 1.5 cm (Figure 6) with a glistening white and fibrofatty outer surface was observed. The cut surface showed an ill-defined, grayish-white area measuring 3 × 2 × 1 cm with focal punctate areas of hemorrhage and necrotic areas. No bony tissue was included. On microscopic examination (Figures 7A, 7B), the sections showed fibrocollagenous tissue with dilated, anastomosing, thin-walled capillaries lined by flattened endothelium. Also seen were several phleboliths obstructing the capillary lumen and hemosiderin-laden macrophages. The vessels were highlighted by CD34 and CD31. Macrophages were highlighted by CD68. No cellular atypia was seen. Histomorphological features were consistent with the diagnosis of synovial hemangioma.

## Discussion

Synovial hemangioma is seen in the adolescent age group commonly in the second decade of life with no gender predisposition [1]. Although more common in the knee joint, it is also reported in the elbow, wrist, and ankle [5]. The pathogenesis is still debatable. Anatomically, it has been divided into intra-articular (within the joint capsule), juxta-articular (outside the joint capsule but in close relation to it), and intermediate (localized intra-articularly but also extra-articular) [4].

The signs and symptoms of synovial hemangioma are non-specific, with a low intensity and long duration of pain and swelling, and minimal limitations in activities of daily living, which delays its presentation. Intra-articular synovial hemangiomas of the knee may cause pain and mechanical symptoms, which may present relatively early [1].

X-rays may reveal no abnormality but seldom show phleboliths within a soft tissue swelling, as in our case, and other non-specific abnormalities such as periosteal thickening and arthritic changes. Contrast-enhanced MRI appears to be the investigation of choice for diagnosis, which also helps in surgical planning by three-dimensional localization of the tumor. Histopathological examination is the keystone in establishing a definitive diagnosis. Histopathologically, they are classified as capillary, cavernous, and mixed or arteriovenous types [3].

Treating these patients in the early phases is advisable, particularly in intra-articular tumors, as it can cause recurrent hemarthrosis, spread through the entire synovium, can lead to joint destruction, and cause direct invasion of surrounding muscles, fat, and vital structures, making it unresectable [5]. Embolization may be indicated in cases of diﬀuse lesions, when complete surgical resection is diﬃcult or impossible, or when the tumor mass is nourished by a large blood vessel [6,7]. Non-surgical treatment and observation have been recommended by some studies in diﬀused cases [8-10]. Open excisional biopsy is more often recommended than arthroscopic excision for most cases as it provides better visualization of the margins. Furthermore, en-bloc resection offers symptomatic relief and potentially, minimal chance of recurrence. Prognosis in the localized form is generally good.

It is essential to differentiate synovial hemangioma from other masses that originate from the synovial membrane, such as pigmented villonodular synovitis, synovial osteochondromatosis, dendritic lipoma, and synovial sarcoma [11], as well as soft tissue chondroma, arteriovenous malformation, and phleboliths.

## Conclusions

Generalized conclusions cannot be drawn by extrapolating a single case, and each case must be worked up and managed accordingly. Although rare, synovial hemangioma must be considered in the differential for persistent joint swellings. Early diagnosis and treatment lead to favorable outcomes, preventing complications such as joint destruction and infiltration into the surrounding tissues. A multimodal approach with due deliberation with the pathologist and radiologist is the keystone in managing musculoskeletal tumors. Histopathological confirmation of diagnosis is crucial. While embolization may be considered for lesions when complete excision is not possible, surgical excision remains the definitive treatment, offering symptomatic relief and a low recurrence rate. A routine follow-up is warranted to monitor for recurrence. This case highlights the importance of considering synovial hemangioma in the differential diagnosis of joint swellings and the role of a multimodal approach in management.
